# DNA methylation biomarkers in stool for early screening of colorectal cancer

**DOI:** 10.7150/jca.34944

**Published:** 2019-08-28

**Authors:** Jie Chen, Haipeng Sun, Weisen Tang, Lin Zhou, Xi Xie, Zhan Qu, Mengfei Chen, Shunyao Wang, Ting Yang, Ying Dai, Yongli Wang, Tangjie Gao, Qiao Zhou, Zhuo Song, Mingmei Liao, Weidong Liu

**Affiliations:** 1 Department of Essential Surgery, Xiangya Hospital, Central South University, Changsha, Hunan, 410008, P. R. China; 2 GeneTalks Biotech Co., Ltd. Changsha, Hunan, 410000, P. R. China; 3 Key Laboratory of Nanobiological Technology of Chinese Ministry of Health, Xiangya Hospital, Central South University, Changsha, Hunan, 410008, P.R. China.

**Keywords:** DNA methylation, colorectal cancer, biomarker, stool

## Abstract

**Objective**: Detection of aberrant methylated genes in feces has been developed as an early screening method for colorectal cancer. The aim of this study was to probe the methylation status of SEPT9, BMP3, NDRG4, and SDC2 in stool and study whether methylation of these genes is associated with colorectal cancer.

**Materials and Methods**: DNAs were isolated and purified from cancerous and non-cancerous stool samples and colorectal cancer tissue. Gene methylation levels were quantified by methylation-specific PCR on SEPT9, BMP3, NDRG4, and SDC2 and analyzed by a diagnostic model.

**Results**: DNA methylation of SEPT9, NDRG4 and SDC2, but not BMP3, had diagnostic potential for detecting colorectal cancer. Moreover, integration of SEPT9, NDRG4, and SDC2 methylation demonstrated high feasibility for detecting colorectal cancer and adenoma, with better performance on colorectal cancer than adenoma.

**Conclusion**: The methylation of SEPT9, NDRG4, and SDC2 in stool may be a potential biomarker for early screening of colorectal cancer.

## Introduction

Colorectal cancer (CRC) is the third most common cancer and the second leading cause of cancer-related deaths in Western countries 1. With the development of the economy and the westernization of diets, the morbidity and mortality of CRC has increased significantly in developing countries 2. For distant metastasis in CRC, the 5-year survival rate is less than 10 %. On the other hand, if CRC can be diagnosed at an early stage, the 5-year survival rate increases significantly 3. Nevertheless, most patients are diagnosed with a late stage of cancer when symptoms appear 4. Therefore, a convenient and effective method for early diagnosis of CRC is necessary.

Currently, the most common diagnostic methods for CRC are colonoscopy and fecal occult blood testing. These methods have the disadvantages of high cost, invasiveness and relatively high risk of complications and therefore fail to satisfy the demand of CRC mass screening. Additionally, to detect early-stage lesions, these tests may need substantial improvement 2. Thus, researchers have begun to investigate methods with low cost, non-invasiveness, and that have high precision in clinical practice, such as stool- and serum-based screening 5. In theory, stool-based screening could be an ideal choice for early detection of CRC, as neoplastic cells are continuously shed into the colonic lumen and mixed with stool. This method requires only a small amount of feces, which is easy to collect without any special restrictions.

For CRC, the main process of benign polyps becoming malignant tumors is the accumulation of genetic and epigenetic alterations that transform colonic epithelial cells into colon adenocarcinoma cells. These cells are continuously shed into colonic lumen and mixed with the stool 6. Aberrant DNA methylation of tumor suppressor genes induces abnormal expression of downstream genes, which is an important step in the process of tumorigenesis. Therefore, genes with methylated DNA that could be detected in stool may have the potential as biomarkers for CRC screening in the clinic. Indeed, aberrant DNA methylations have been found correlated with CRC 7. For example, aberrant methylation of N-Myc downstream regulated gene 4 (NDRG4) and bone morphogenic protein 3 (BMP3) could be used for CRC screening 8. Moreover, aberrant methylation of septin 9 (SEPT9) and syndecan 2 (SDC2) has been probed in stool or plasma of CRC patients 9-11.

The aim of this study was to test and verify that detecting DNA methylation of genes in stool could reveal biomarkers for early detection of CRC. We examined the associations between the methylation status of NDRG4, BMP3, SEPT9 and SDC2 and CRC.

## Materials and Methods

### Sample collection

Tissue samples were from patients with CRC with informed consent from Xiangya Hospital of Central South University. Ethics approval was given by the medical ethics committee of Xiangya Hospital of Central South University (reference no.: 201712844). The methylation status of genes was analyzed in matched patient tissue samples (n=23 patients) from tumor, non-tumor adjacent tissue, and normal tissue.

Stool samples (about 5 g) were collected from CRC patients and healthy individuals with informed consent from Xiangya Hospital. Stool samples were kept in 50-mL tubes with 15 mL preservative buffer (0.5 mol/L Tris, 0.15 mol/L EDTA, 10 mmol/L NaCl, pH 9.0). Once collected, samples were immediately stored at -80 ˚C. The status of patients for all samples-CRC, adenoma, and normal healthy stool-was confirmed by colonoscopy or histology.

This study was approved by the Institutional Review Board at Xiangya Hospital.

### DNA Isolation

For tissues, DNA was isolated by using the QiaAMp DNA Mini kit (QIAGEN, Hilden, Germany) according to manufacturer's instructions. Stool samples were homogenized in preservative buffer with a shaker device. After homogenization, the sample was centrifuged at 4000 g for 15 min. A 10-mL amount of supernatant was transferred into a new tube, and 10 mL lysis buffer (GenMagBio, Beijing) was added into the supernatant and incubated at 55 ˚C for 20 min. A 2-mL amount of 10% polyvinylpolypyrrolidone was added and incubated with a mixer at room temperature for 30 min. Then, the sample was centrifuged at 4000g for 15 min, and the supernatant was transferred into a new tube. A 30 μL amount of Acryl Carrier, 60 μL Magnetic Beads (GenMagBio, Beijing) and 240 μL proteinase K was added into this tube and incubated at room temperature for 30 min. The tube was placed on a Magnet until the solution cleared and the beads were pelleted against the magnet. The supernatant was discarded and beads were washed with wash buffer twice, then eluted with 60 μL distilled water. The DNA concentration was measured by using NanoDrop 2000 (Thermo Scientific, MA, USA). All purified DNA was stored at -20 °C.

### Bisulfite treatment and DNA purification

A commercial kit EZ DNA Methylation-Gold kit (ZYMO Research, CA, USA) was used according to the manufacturer's instructions. Briefly, genomic DNA was first denatured at 98 °C for 10 min, then treated with sodium bisulfite at 65 °C for 2.5 hr. Then, DNA was added into a spin column and desulphonated by adding desulphonation buffer and incubated at room temperature for 20 min. Bisulfite-converted DNA was purified by using a spin column and eluted with 20 μL distilled water. The purified DNA was used immediately or stored at -20 °C.

### Quantitative methylation-specific PCR (qMSP)

qMSP was used to quantify methylation levels of SEPT9, BMP3, NDRG4, and SDC2. Specific primers for the promoter region of target genes were designed, and the ACTB gene was used as a reference for bisulfite-converted DNA input. Primers and probes used are in Table 3. Briefly, 5 μL bisulfite-converted DNA was used as a template, and the total PCR reaction volume was 30 L: 300 nM each primer, 200 nM each probe, and 15 μL AmpliTaq Gold 360 Master Mix (AB applied Biosystems, CA, USA). The PCR reaction was performed with StepOne plus (Thermo Scientific, MA, USA) at 95˚C for 10 min; 45 cycles at 95˚C for 15 sec, 60˚C for 30 sec; 20˚C for 2 min. The level of methylation of each gene was calculated as 2 to the power of qMSP Ct difference between the reference gene and target gene.

### Diagnostic model building

The diagnostic model was built by fitting different genes into a binary logistic regression model (glm package, R). For a single gene marker, the target gene and reference gene ACTB were used for fitting. The optimal threshold was determined by Youden's J index. To confirm the performance of models, we randomly divided the whole cohort into two parts: training cohort (60%) and testing cohort (40%). The training cohort was used for model building and the testing cohort for evaluating the performance of the models.

### Statistical analysis

Wilcoxon rank sum test was used to compare methylation levels between different groups. Receiver operation curve (ROC) and area under the ROC curve (AUC) values were estimated to evaluate the accuracy of diagnosis, and the Delong's test was used to compare differences in AUC values (pROC). The plot of the model effect and coefficients involved using jtools. All statistical analyses involved use of R v3.5.0. P <0.05 was considered statistically significant.

## Results

The characteristics of patients with CRC tissue samples are in **Table 1**. Stool samples were collected from 41 patients with CRC, 37 patients with adenoma, and 152 normal healthy individuals. Their characteristics are in **Table 2**.

### Methylation status of different biomarkers in colorectal tissues

**Fig 1:**
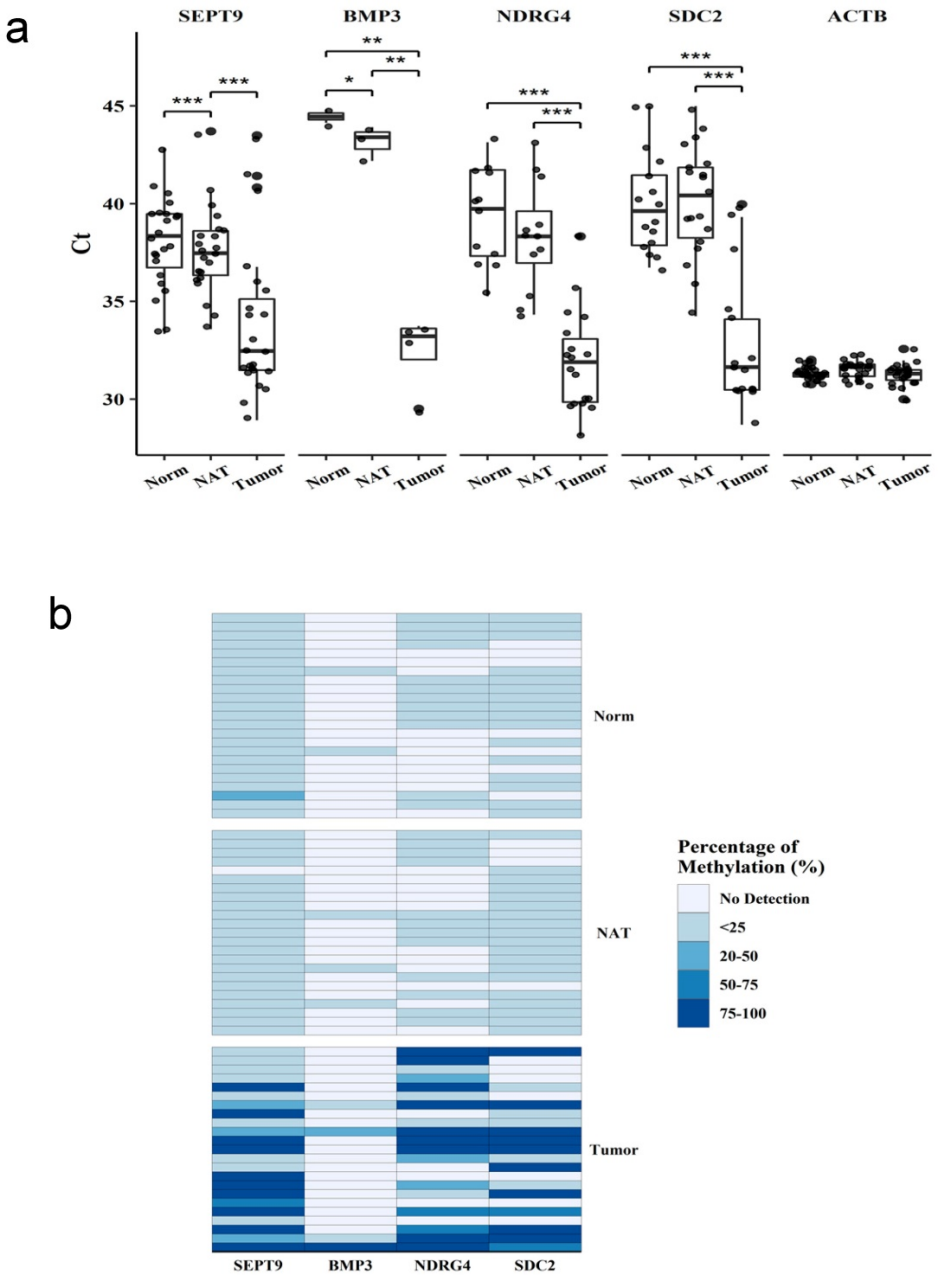
** Methylation in tissue samples from patients with colorectal cancer.** Methylation of four genes in tissue samples. Methylation levels of each gene were measured by qMSP in samples from normal tissue (Norm), non-tumor adjacent tissue (NAT), and tumor tissue (Tumor). (a) Ct values for each gene in different samples. (b) Methylation level represent by ratio to the reference gene ACTB. Data are mean±SD. *P<0.05, **P<0.01, ***P<0.001

The methylation status of SEPT9, BMP3, NDRG4, and SDC2 was analyzed by using qMSP in matched patient tissue samples (n=23) from tumor, non-tumor adjacent tissue, and normal tissue (Figure 1a). The targeted genes showed significantly lower Ct values in tumors than in normal or non-tumor adjacent tissues, indicating the higher frequency of methylation in tumor. The methylation level was presented as the difference in Ct between the targeted gene and the reference gene (Figure 1b). NDRG4 and SDC2 had higher frequency and level of methylation in tumors than in normal or non-tumor adjacent tissues. SEPT9 had high frequency of methylation in all three tissue types, however, the level of methylation was significantly higher in tumor tissues. On contrary, the frequency and level of methylation was much lower for BMP3 than other genes in all three tissue types. Therefore, SEPT9, NDRG4 and SDC2, but not BMP3, had diagnostic potential for detecting CRC.

### DNA methylation status in stool

**Fig 2:**
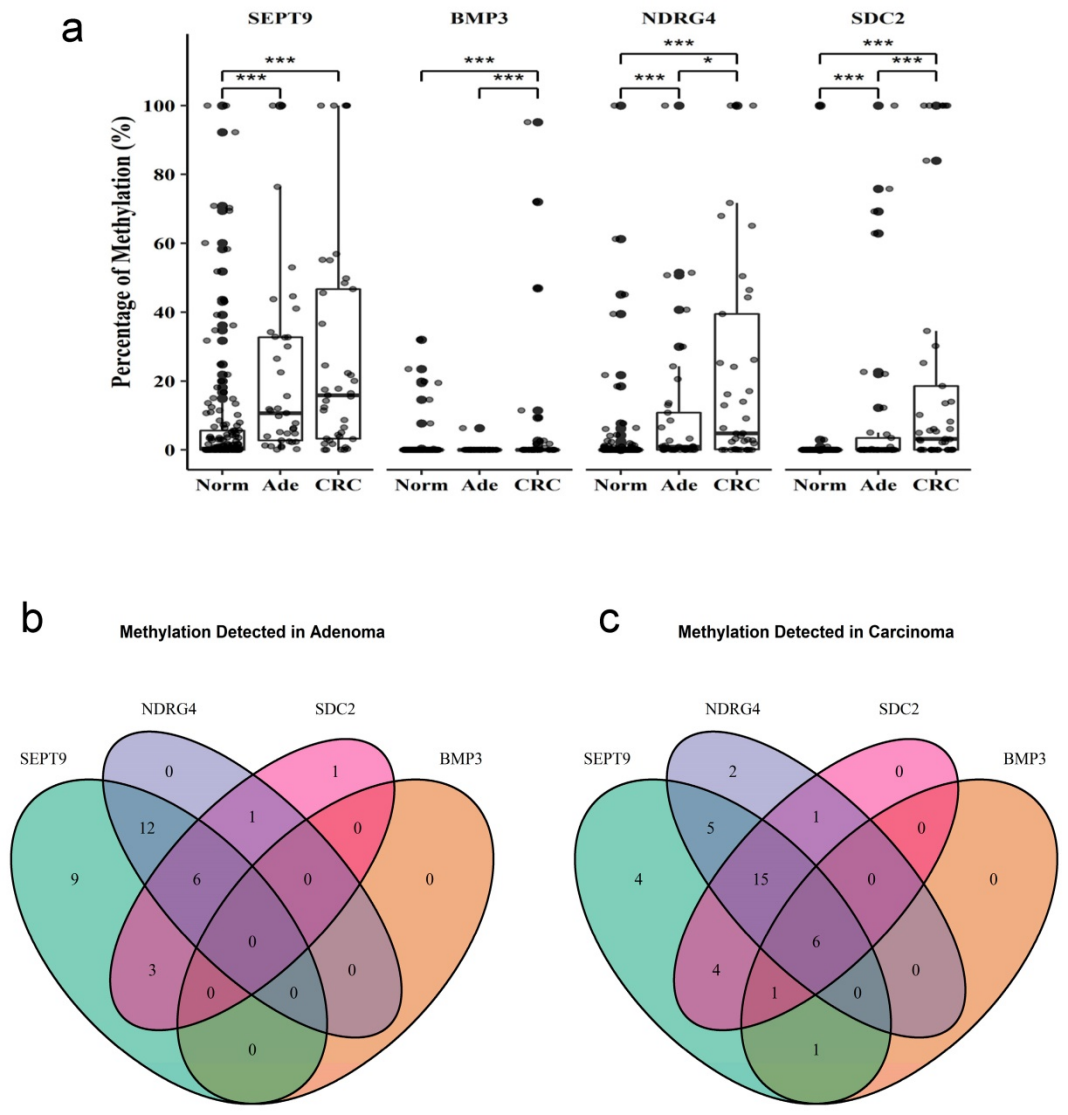
** Methylation of four genes in stool samples from patients with and without colorectal cancer.** Methylation levels of each gene were measured by qMSP in (a) 41 samples of (CRC), 37 of adenoma (Ade), and 152 of normal (Norm) stool. The intersection of each gene detected in (b) adenoma or (c) CRC samples. Data are mean±SD. *P<0.05, ***P<0.001

The methylation level of SEPT9, BMP3, NDRG4, and SDC2 in stool samples was quantified by using qMSP. All four genes showed a significantly higher level of methylation in adenoma or CRC stool than normal stool (Figure 2a). However, the frequency of BMP3 methylation was much lower compared to the three other genes. BMP3, NDRG4, and SDC2 showed a significantly higher methylation level in CRC than adenoma samples. This result suggested that methylation level was associated with the severity of lesions. Moreover, SEPT9 methylation was discovered in most CRC and adenoma samples. Only two samples had NDRG4 hypermethylation, only one sample had SDC2 hypermethylation, and no sample had BMP3 hypermethylation (Figure 2b).

### Performance of biomarkers in DNA test in stool

**Fig 3:**
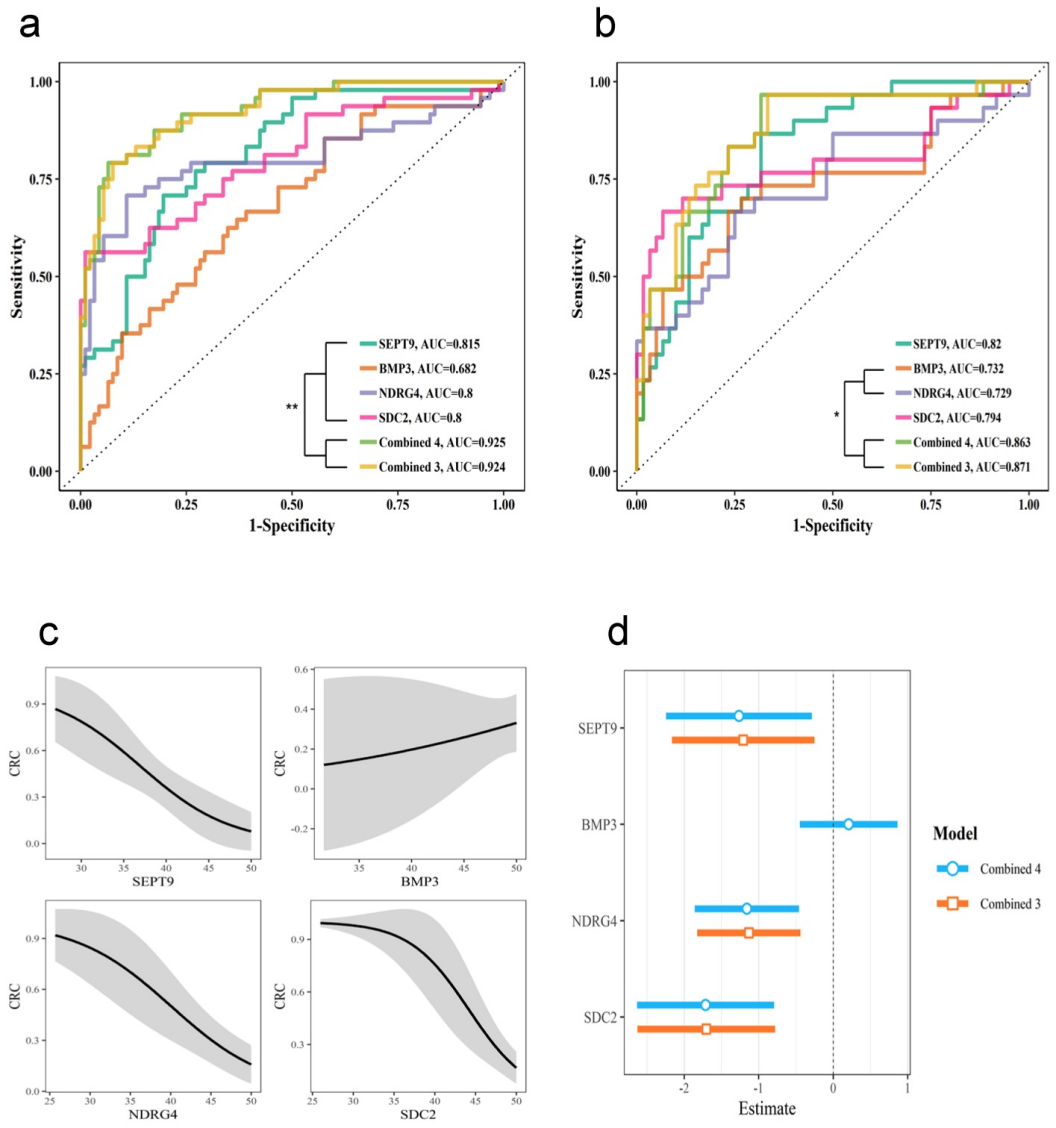
** Diagnostic test with different genes in stool samples.** The diagnostic performance with individual genes and the combination of four genes (Combine 4) or three genes, SEPT9, NDRG4, and SDC2 (Combine 3). Receiver operating characteristic (ROC) curves for training cohort (a) and testing cohort (b). (c) Effect of each gene in the Combine 4 model. (d) Comparing the coefficients for the Combine 4 and Combine 3 models.

The whole stool samples were divided into two sets: a training set (normal n=92, adenoma n=23, CRC n=25) and a testing set (normal n=60, adenoma n=14, CRC n=16). The training set was used to build logistic regression models for each gene, the combination of the three genes SEPT9, NDRG4, and SDC2 (Combined 3) and the combination of all four genes (Combined 4). The ROC curve for each model was plotted (Figure 3a). The AUC was significantly higher for the combined models than for the individual gene models. Moreover, the Combined 4 model had similar AUC as the Combined 3 model, which suggests that BMP3 had very limited contribution to detection accuracy.

We then used these models with the testing cohort; the resulting ROC curve is shown in Figure 3b. The detection performance was still higher with the combined models, and the Combined 3 model performed similarly to the Combined 4 model. The effect of the Combined 4 model in Figure 3c showed that BMP3 had relative low slope and huge confidential intervals. Compared to the Combined 4 and Combined 3 models (Figure 3d), models for SEPT9, NDRG4, and SDC2 had very similar coefficients, but BMP3 had a coefficient close to zero. All these results indicated that the combination of SEPT9, NDRG4, and SDC2 methylation levels was sufficient for effective detection of CRC and adenoma.

### Different performance in CRC and adenoma

**Fig 4:**
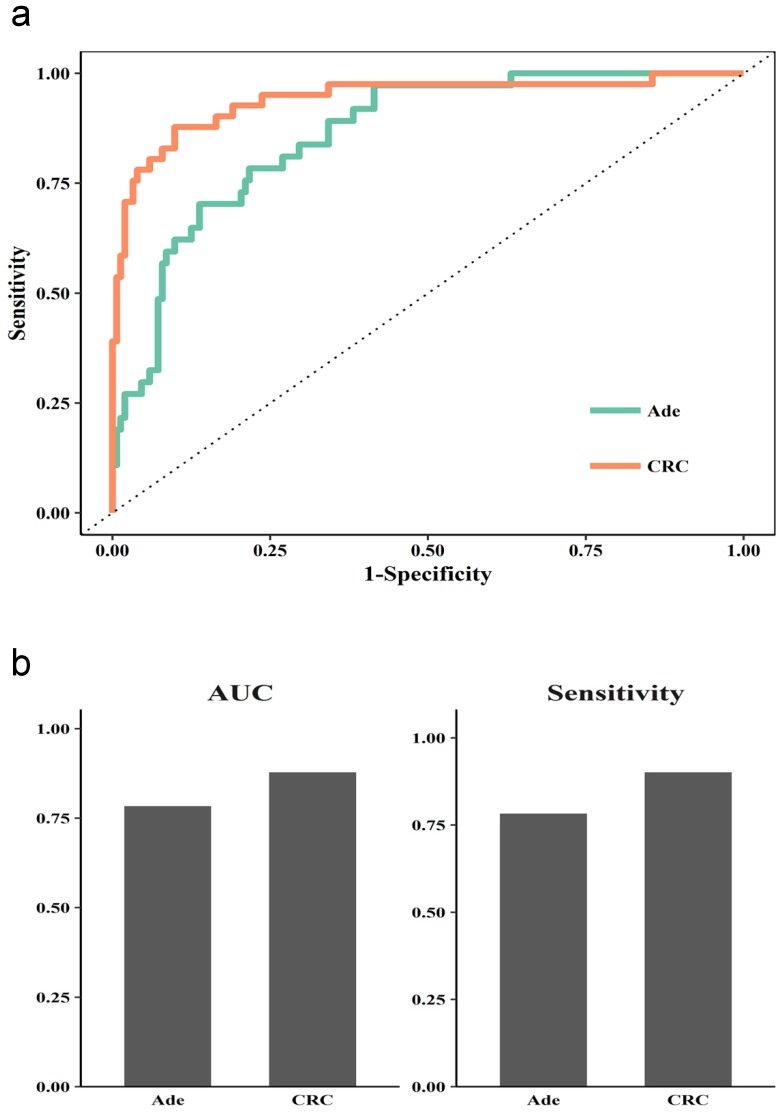
** Diagnostic performance of the Combined 3 model in stool for CRC and adenoma.** (a) ROC curves for normal vs adenoma sample (Ade) or CRC sample. (b) AUC and sensitivity for the diagnostic performance in Ade and CRC samples.

We further checked the performance when combining methylated SEPT9, NDRG4, and SDC2 for detecting adenoma and CRC. The ROC curve suggested a better performance for detecting CRC than adenoma (Figure 4a). The AUC was higher for CRC than adenoma (0.88 vs 0.78), and the sensitivity was higher (0.9 to 0.78).

## Discussion

Detection of aberrant methylated genes in feces has been found an early screening method for CRC. In this study we aimed to detect the methylation status of SEPT9, BMP3, NDRG4 and SDC2 in human stool and whether methylation of these genes associates with CRC. We found that DNA methylation of SEPT9, NDRG4 and SDC2, but not BMP3, may have diagnostic potential for detecting CRC. Methylation of SEPT9, NDRG4 and SDC2 combined has high feasibility for detecting CRC and adenoma, with better performance for detecting CRC over adenoma. Detecting methylated SEPT9, NDRG4, and SDC2 in stool may be a biomarker for early screening of CRC.

CRC is one of the most common malignancies 12. In the clinic, advanced CRC always means poor prognosis. Therefore, early diagnosis and treatment can help improve the potential for surgery dissection and the prognosis 13, 14. In recent years, because of the ease and feasibility of fecal gene detection, a number of genes have been selected as biomarkers for CRC screening 15. Methylated SEPT9, TWIST1, IGFBP3, GAS7, ALX4 detected in stool were found correlated with CRC 16. However, less than 0.01% of DNA is in human stool and identifying these trace amounts of methylated genes from wild-type DNA is challenging 17. In the past few years, significant progress has been made on the sensitivity and specificity of biomarker in stool from CRC patients, demonstrating it as a viable option for CRC screening 18-22. Ahlqist and colleagues reported that during the progression of CRC tumorigenesis, biomarkers release into the stool earlier than into blood 23. Thus, aberrant methylated biomarker screening in stool may help with CRC prevention and early detection.

In this study, we demonstrated that SEPT9, NDRG4, and SDC2 had higher frequency and level of methylation in tumors than in normal or non-tumor adjacent CRC tissues, indicating that these methylated genes may have diagnostic potential for CRC screening. However, BMP3 had very limited contribution to detection accuracy in stool samples. Furthermore, the combination of methylated SEPT9, NDRG4, and SDC2 showed high feasibility of detection of CRC and adenoma and further study showed better performance in detecting CRC than adenoma.

DNA methylation is one of the most important factors driving the occurrence of CRC 24-26. An increasing number of genes with methylation are associated with the tumorigenesis of CRC 27. DNA methylation is a kind of chemical modification induced by methyl transferases, which regulates transcription without altering DNA primary sequence 28. Carmona and colleagues demonstrated that methylated AGTR1, WNT2 and SLIT2 could be biomarkers for a non-invasive diagnosis of CRC 6. Methylated NDRG4 in stool was found associated with CRC 29. Afsaneh et al. showed that SFRP1 and SFRP2 methylation had promising accuracy for detecting CRC as well as an early stage of cancer, adenoma 23. All these studies have supported the identification of DNA methylation markers in stool as an ideal method for early screening of CRC with high sensitivity and specificity.

Aberrant methylated genes, such as SEPT9, BMP3, NDRG4, and SDC2, are strongly associated with CRC 9, 30-34, and methylation usually occurs in the early stages of the disease. In this study, we detected the methylation status of SEPT9, BMP3, NDRG4, and SDC2 in both CRC tissue and stool. In CRC tissue, DNA methylation of SEPT9, NDRG4, and SDC2 but not BMP3 had diagnostic potential for detecting CRC. In stool samples, the methylation level of the four candidate genes was related to the severity of lesions. These data provide a theoretical basis for showing that these genes can be used as biomarkers for CRC screening.

In summary, our results suggest that methylated genes detected in stool DNA could be biomarkers for early screening of CRC. Three genes (SEPT9, NDRG4, and SDC2) showed a higher level and frequency of methylation in stool and could be the candidate biomarkers for stool-based screening, especially when combined. Furthermore, it is possible for a combined of this multitarget DNA stool test to become commercially available and provides a new non-invasive choice for diagnosis of CRC and adenoma. However, the limitations in this study include a relatively small number of specimens and age differences between groups. To reduce costs, the detection points are not extensive enough. A larger study is needed to further examine the role of methylated DNA in stool for CRC screening.

## Figures and Tables

**Table 1:** Clinicopathological characteristics for participants with tissue samples used in this study (n=23)

Characteristics of participants	
Sex	
Male	12 (52)
Female	11 (48)
Age, years, mean (range)	57.5 (39-81)
Tumor location	
Colon	9 (39)
Rectum	14 (61)
Stage	
I-II	17 (74)
III-IV	6 (26)

Data are n (%) patients unless indicated.

**Table 2:** Clinicopathological characteristics for participants with stool samples used in this study (total n=230)

Characteristics of participants	
Healthy control (n=152)	
Sex (%)	
Male	84 (55)
Female	68 (45)
Age, years, mean (range)	46.2 (21-74)
Adenoma (n=37)	
Sex (%)	
Male	23 (62)
Female	14 (38)
Age, years, mean (range)	56.7 (41-80)
CRC (n=41)	
Sex (%)	
Male	19 (46)
Female	22 (54)
Age, years, mean (range)	55.9 (28-73)

Data are number (%) of patients unless indicated.

**Table 3:** Primers and probes used in this study

Name	Sequence 5' - 3'	Description
SEPT9_F	TATTAGTTATTATGTCGGATTTCGC	SEPT9 forward primer
SEPT9_R	TCCAACACGTCCGCGACCGCA	SEPT9 reverse primer
BMP3_F	TTTGAAAATATTCGGGTTATATACGTCGC	BMP3 forward primer
BMP3_R	ATAAACTCTTCCCCAACAACTACGCGAA	BMP3 reverse primer
NDRG4_F	ATCGATCGGGGTGTTTTTTAGGTTTC	NDRG4 forward primer
NDRG4_R	GCCTTCTACGCGACTAAAATACCCGAT	NDRG4 reverse primer
SDC2_F	GGGAGTGTAGAAATTAATAAGTG	SDC2 forward primer
SDC2_R	TCCCAACCRCTACTTACAA	SDC2 reverse primer
ACTB_F	GAAAGGGTGTAGTTTTGGGAGGTTAG	ACTB forward primer
ACTB_R	AATAACCCAAATAAATAACCCACTACCTC	ACTB reverse primer
SEPT9_P	AACGCGTAGTTGGATGGGATTATTTCGGA	SEPT9 probe
BMP3_P	AGCGTTGGAGTGGAGACGGCGTTCGTAGCGT	BMP3 probe
NDRG4_P	CGTCGCGGTTTTCGTTCGTTTTTTCGTTCGT	NDRG4 probe
SDC2_P	GCGAGCGCCCCCGAGCCCCG	SDC2 probe
ACTB_P	CCTCTTCTAATAACCACCTCCCTCCTTCCTAAC	ACTB probe
